# Supplementary material for: An atlas of O-linked glycosylation on peptide hormones reveals diverse biological roles
Source: Nat Commun. 2020 Aug 20;11:4033. doi: 10.1038/s41467-020-17473-1 (PMC7441158; doi:10.1038/s41467-020-17473-1)
Supplement: Supplementary file 1 — Supplementary Information [file 41467_2020_17473_MOESM1_ESM.pdf]

Supplementary information

An atlas of O-linked glycosylation on peptide hormones reveals wide biological roles

Madsen *et al.*

Supplementary Figure 1

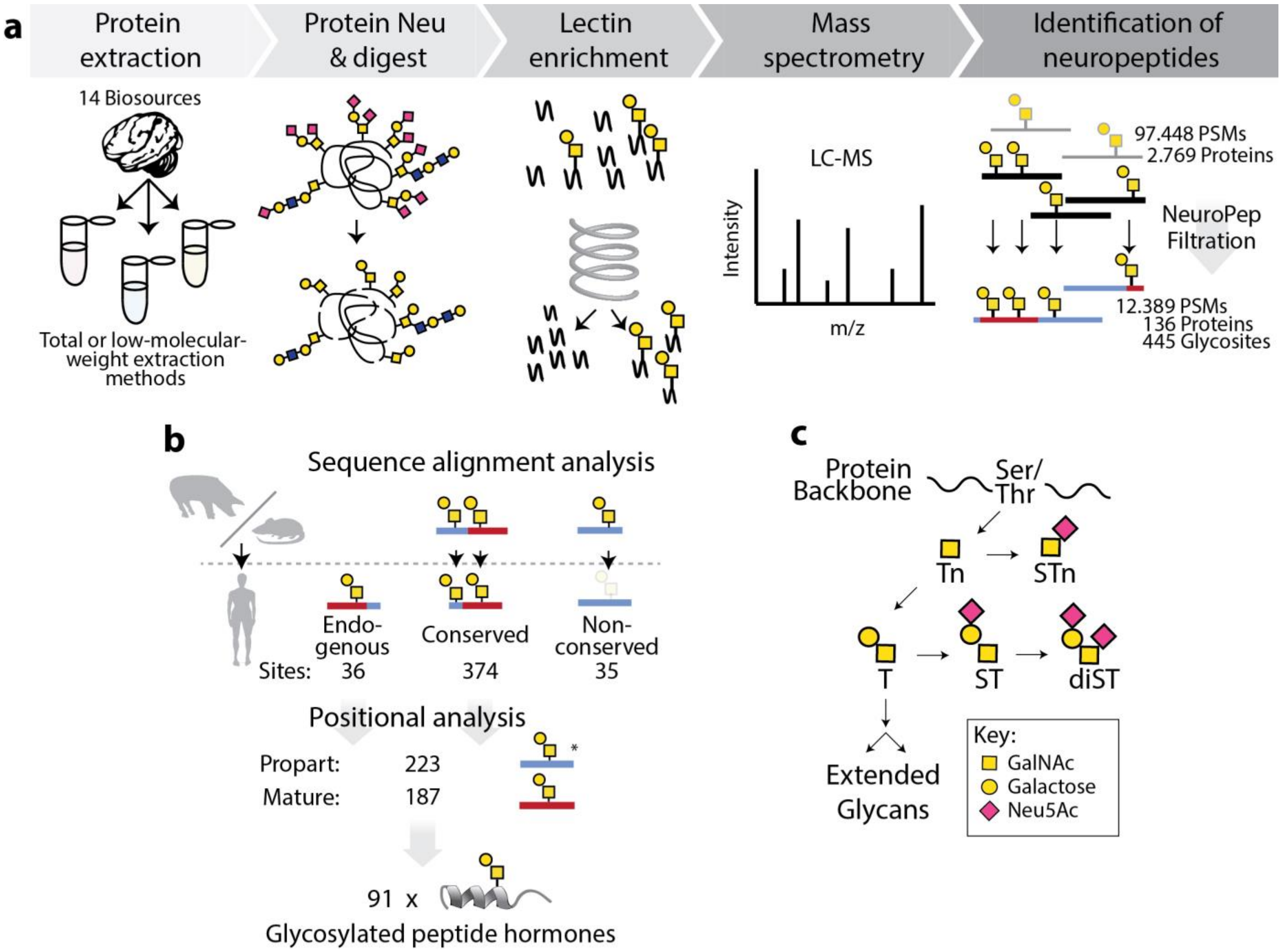

Supplementary Figure 1. Workflow for identification of glycosylated peptide hormones. **a)** Proteins from biofluids (cerebrospinal fluid, plasma), neuroendocrine tissue (brain, cerebellum, pancreas, ileum, heart and prostate) and neuroendocrine cell lines (STC-1, N2a) were extracted using up to three different extraction procedures per sample (see Supplementary Data 3). Subsequently, proteins were reduced, alkylated and digested with either trypsin, GluC or chymotrypsin followed by de-sialylation using neuraminidase and glycopeptide enrichment by LWAC using either PNA, JAC or VVA lectins. Glycopeptides were fractionated using either isoelectric focusing or high pH-fractionation before separation and sequencing by LC-MS/MS. The resulting O-glycoproteome was matched against the NeuroPep database and resulted in identification of 445 glycosites on peptide hormones from all species analysed. **b)** Schematic representation of the data analysis of glycosylated peptide hormones. Using sequence alignment, 374 glycosites in peptide hormone orthologs (pig, rat and mouse) where predicted to be conserved in humans based on the conservative preservation of Ser/Thr residues within +/- 5 amino acids. In total 36 human and 374 conserved glycosites were included for further analysis. Mapping glycosites to full peptide hormone precursor sequences revealed that 223 glycosites were located in pro-domains and 187 glycosites were located on mature peptide hormones. In total, 91 mature peptide hormones contained at least one glycosite (see Supplementary Data 1). *) A large fraction of glycosites located in pro-domains (181 out of 223) are found on the granin, nucleobindin and kininogen superfamilies. **d)** Schematic depiction of the biosynthetic pathway of the most common O-GalNAc-type glycans present on proteins. The majority of circulating O-glycoproteins carry the sialyl-T (ST) and di-sialyl-T (diST) structures. PSM: Peptide-spectrum match.

Supplementary Figure 2

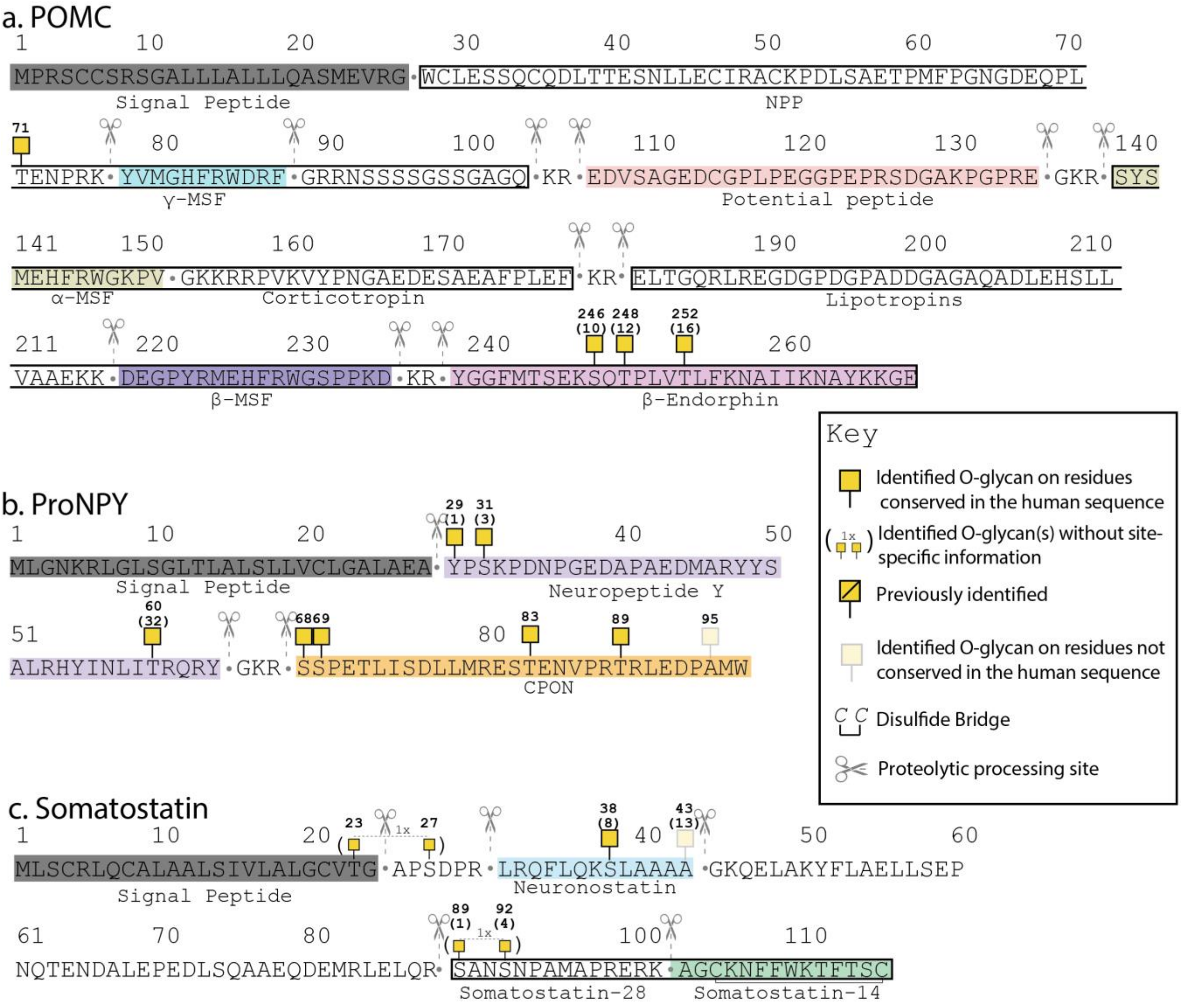

Supplementary Figure 2. Glycosite location in relation to PC processing sites. Schematic illustration of selected peptide hormone precursors with glycosylation sites in close proximity to proteolytic activation sites for **a)** POMC, **b)** proNPY and **c)** prosomatostatin (The Neuronostatin peptide hormone, which is not annotated in the NeuroPep database, was retrieved from the Uniprot database (Uniprot ID: P61278)). Mature peptide hormone glycosite amino acid numbering is shown in parenthesis. Please see supplementary data 1 for peptide hormone abbreviations.

Supplementary Figure 3

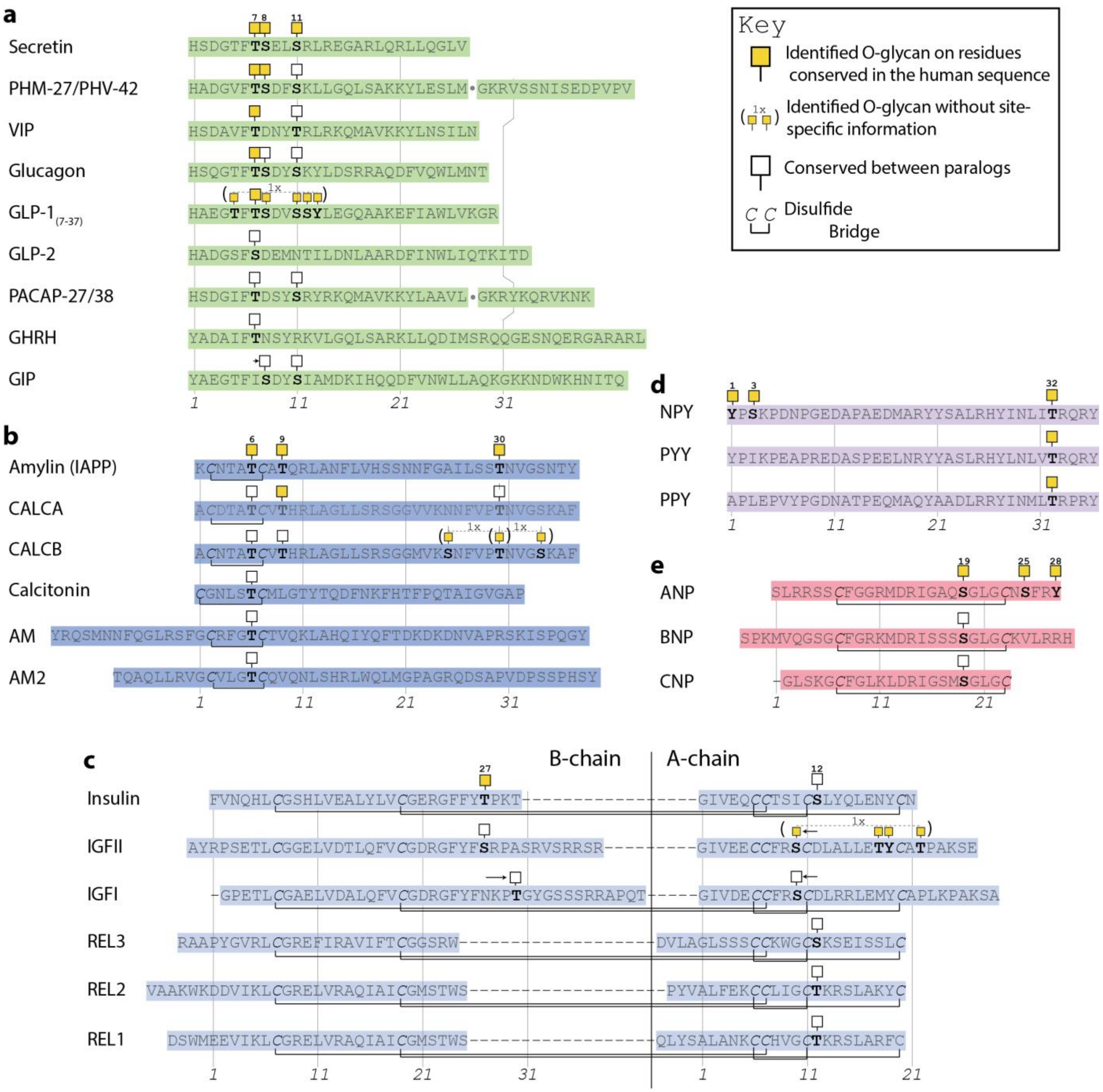

Supplementary Figure 3. Illustration of select O-glycosylated human peptide hormone family sequences. Multiple sequence alignment analysis of the **a)** Glucagon-, **b)** Calcitonin-, **c)** Insulin-, **d)** Neuropeptide Y- and **e)** Natriuretic peptide-families with identified, predicted and conserved O-glycosylation sites shown.Mature peptide hormone sequences are shown. Yellow squares indicate identified glycosylation sites in one or more species investigated. All sites within these families were conserved between the species investigated. White square indicates conserved residues that are confirmed glycosylated in a paralog peptide hormone. Yellow square in parenthesis illustrate ambiguously assigned sites. For GLP-1, a low confidence ETD spectrum suggests the glycan to be positioned on Thr7 (see extracted spectrum 28 in Supplementary Data 4). Please see supplementary data 1 for peptide hormone abbreviations.

Supplementary Figure 4

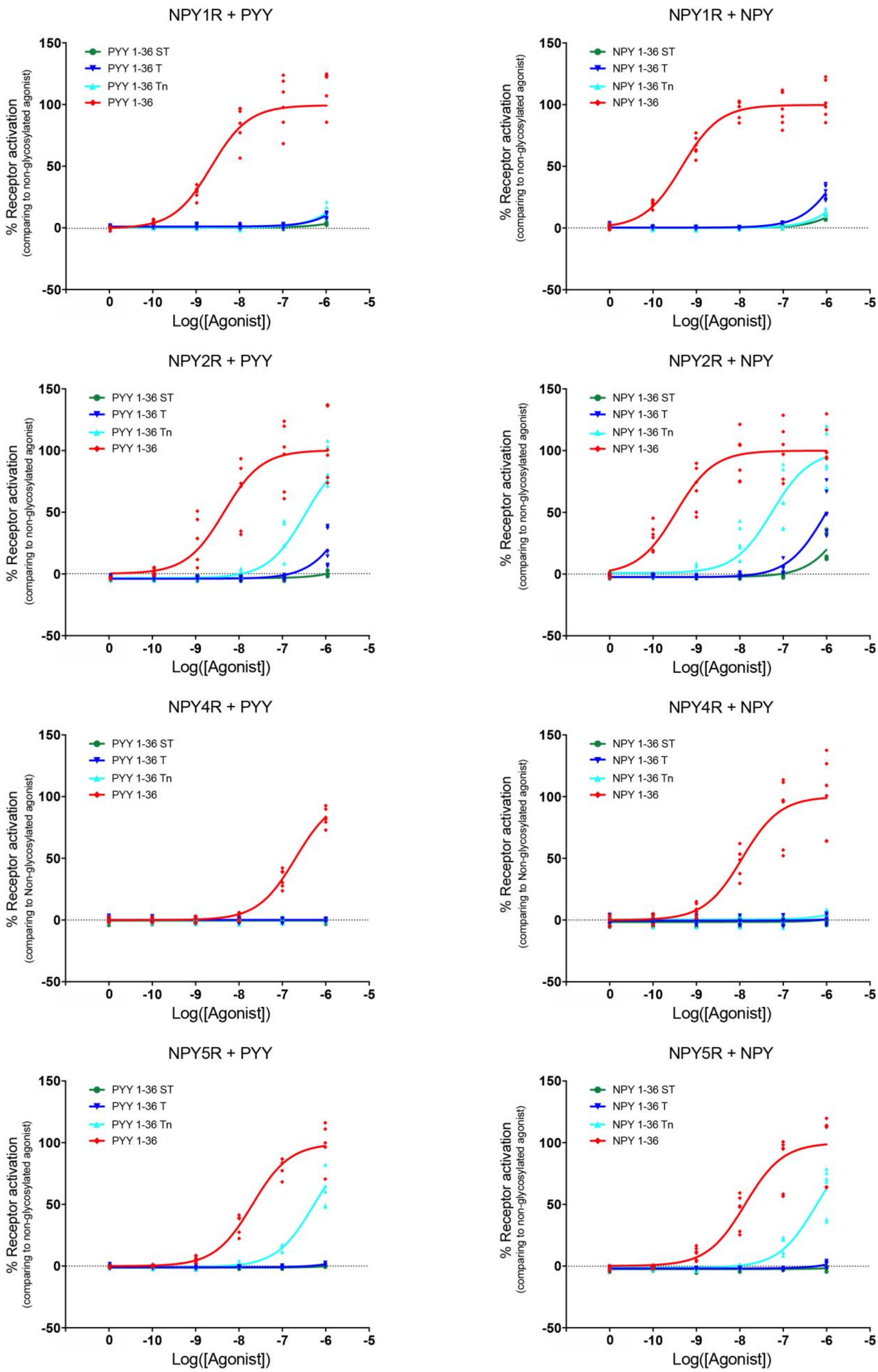

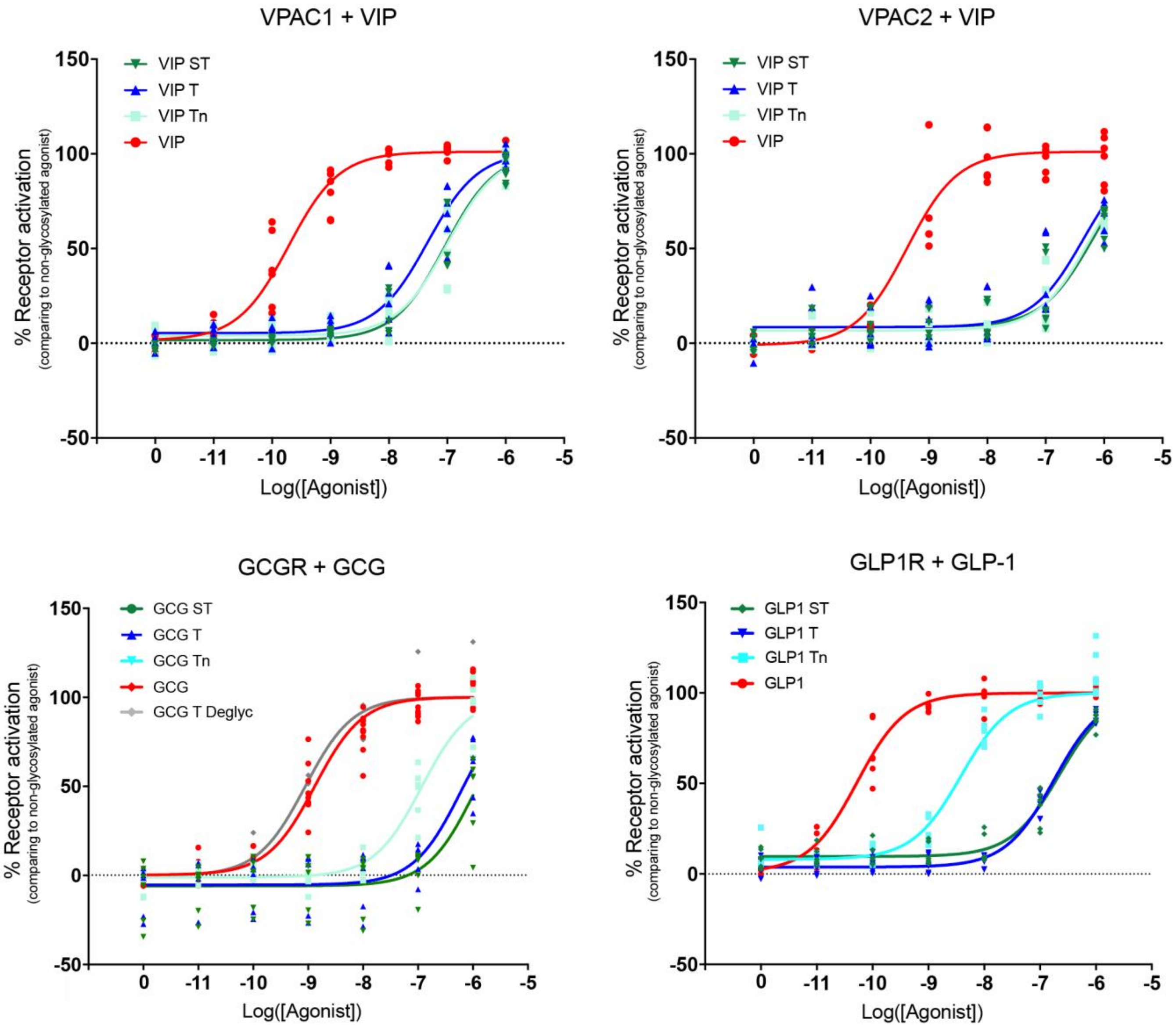

Supplementary Figure 4. Dose-response curves of all tested glycosylated and non-glycosylated peptide hormones. Curves were fitted by non-linear regression (three parameter logistic fit with hill coefficient = 1, top constrained to 100%) calculated using the GraphPad Prism software. All experiments were performed in COS-7 cells transiently transfected with the indicated receptor. 100% receptor activation is defined as the maximal response (E_{max}) obtainable with the cognate non-glycosylated agonist. Individual data points are shown from three independent experiments performed in duplicate assays (apart from deglycosylated GCG T that was done in only one experiment and GCG non-glycosylated that was done in 5 independent experiments). Source data is provided as a Source Data file.

Supplementary Figure 5

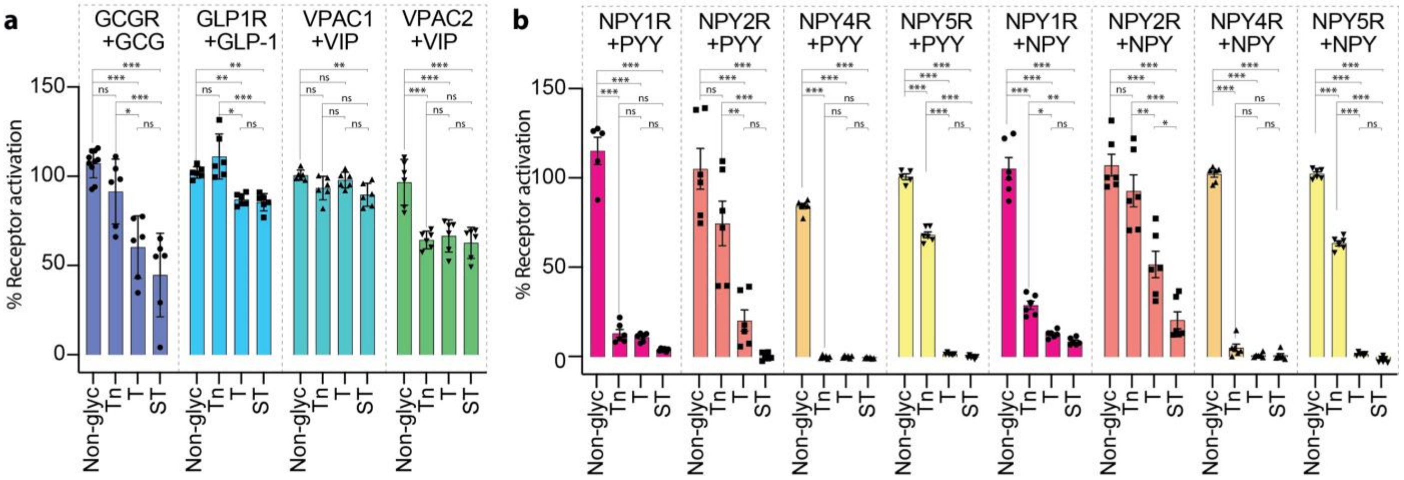

Supplementary Figure 5. Receptor activation using cognate peptide hormones and their glycoforms at a fixed concentration of 1 µM. a) Cell-based receptor activation assay with VIP, Glucagon and GLP1 and their Thr7 glycosylated forms. 100% receptor activation is defined as the maximal response (E_{max}) obtainable with the cognate non-glycosylated agonist. Data points are shown as mean +/- S.E.M. from three independent experiments performed in duplicate assays (apart from non-glycosylated GCG which was performed in 5 independent experiments). **b)** Receptor activation of NPY and PYY and their Thr32 glycoforms performed as described for a)**.** *p < 0.05; **p <0.01; ***p <0.001 (all significant p-values are <.0001 except for the following: **a)** GCGR1-GCG^{Tn}/GCG^{T} p=.0159; GCGR-GCG^{Tn}/GCG^{ST} p=.0003; GLP1R-GLP1/$GLP1^{T}$ p=.0072; GLP1R-GLP1/$GLP1^{ST}$ p=.0034; VPAC1-VIP/VIP^{ST} p=.0097; **b)** NPY1R-NPY^{Tn}/NPY^{T} p=.0147; NPY1R-NPY^{Tn}/NPY^{ST} p=.0021; NPY2R-PYY^{Tn}/PYY^{T} p=.0019; NPY2R-NPY^{Tn}/NPY^{T} p=.0023; NPY2R-NPY^{T}/NPY^{ST} p=.0229) two-tailed one way ANOVA with Tukey's post-hoc test. Source data for panel a) and b) are provided as a Source Data file.

Supplementary Figure 6

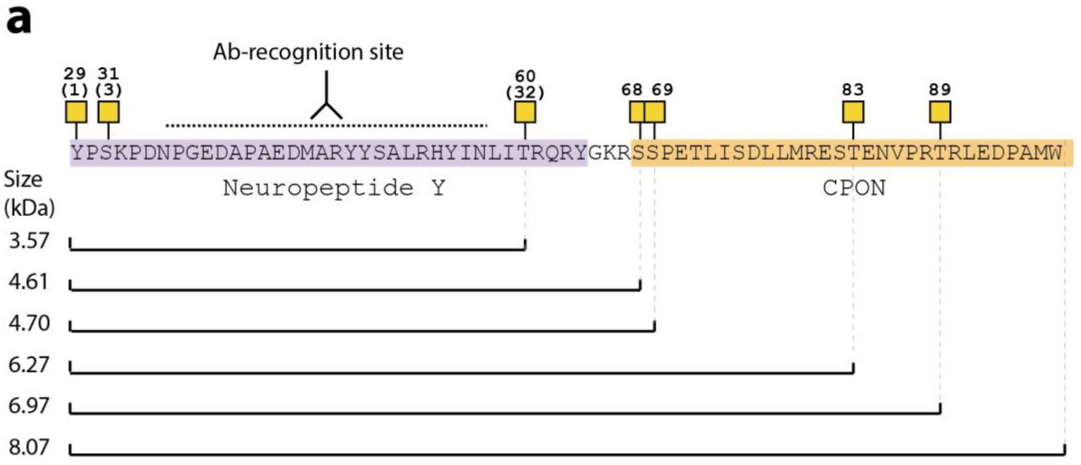

Supplementary Figure 6. Schematic illustration of pro-NPY indicating antibody recognition site and theoretical masses (kDa) of the fragments produced by OpeRATOR cleavage at the N-terminal site of O-glycosites identified in this study (CPON: $Ser^{68/69}$, Thr^{83} and Thr^{89} and NPY: Tyr^{29}, Ser^{31} and Thr^{60} (Mature sites 1, 3 and 32)) of which only Thr^{60} is conserved among the three family members (NPY, PYY and PPY). Both glycosylation of Thr^{60} in NPY and $Ser^{68/69}$ in CPON would result in an immunoreactive fragment of approximately 4-5 KDa after OpeRATOR digestion (Fig. 4g). CPON: C-flanking peptide of NPY.

Supplementary Figure 7

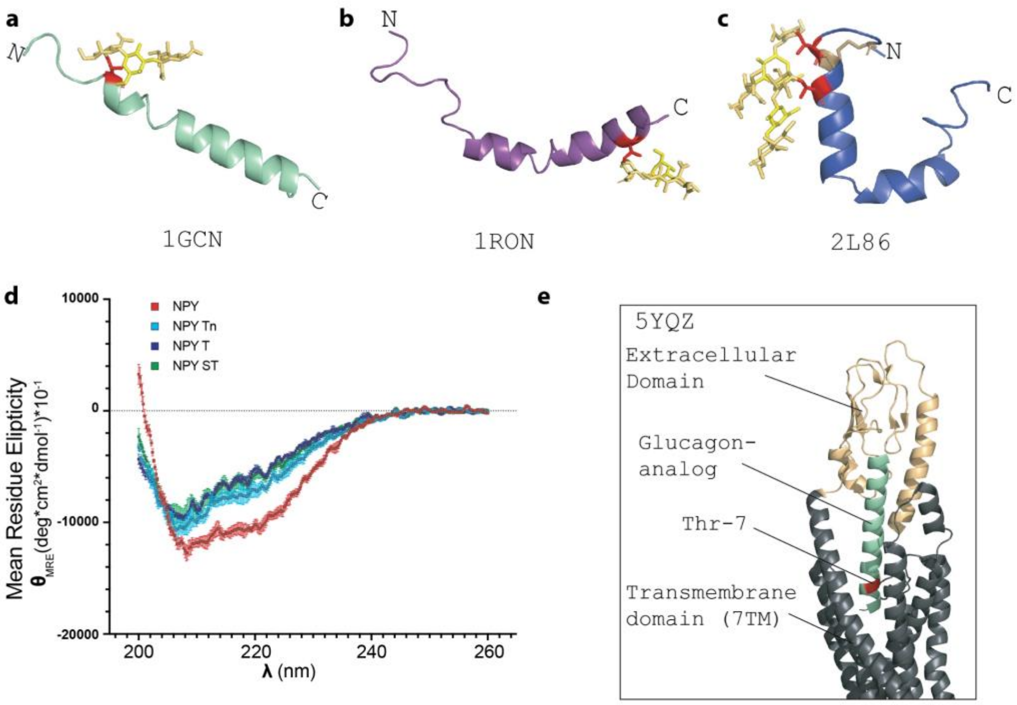

Supplementary Figure 7. Glycans are positioned such that amphipatic alpha-helix folding is disrupted. The conserved glycosites are positioned at the border of the amphipatic helices of **a)** the glucagon family represented by the structure of glucagon (PDB: 1GCN), **b)** The NPY family represented by the structure of NPY (PDB: 1RON) and **c)** the Calcitonin family represented by the structure of Islet Amyloid Polypeptide (PDB: 2L86). The conserved glycosylated residue Thr6 is shared by all members of the calcitonin family, and Thr9 is conserved between calcitonin, and calcitonin gene-related peptides. **d)** CD spectroscopy of NPY or different glycovariants of NPY at 25°C pH 7 in aqueous solution. The rise in ellipticity at 222 nm reflect disruption of the alpha-helix. Ellipticity is expressed as the mean-residue molar elipticity. Mean +/- S.E.M. is shown (n=4 for non-glyc., T and ST; n=3 for Tn). **e)** Structure of the Glucagon receptor with highlight (red) of Thr7 in a glucagon-analog binding to the receptor (PDB: 5YQZ). Source data for panel d) is provided as a Source Data file.

Supplementary Figure 8

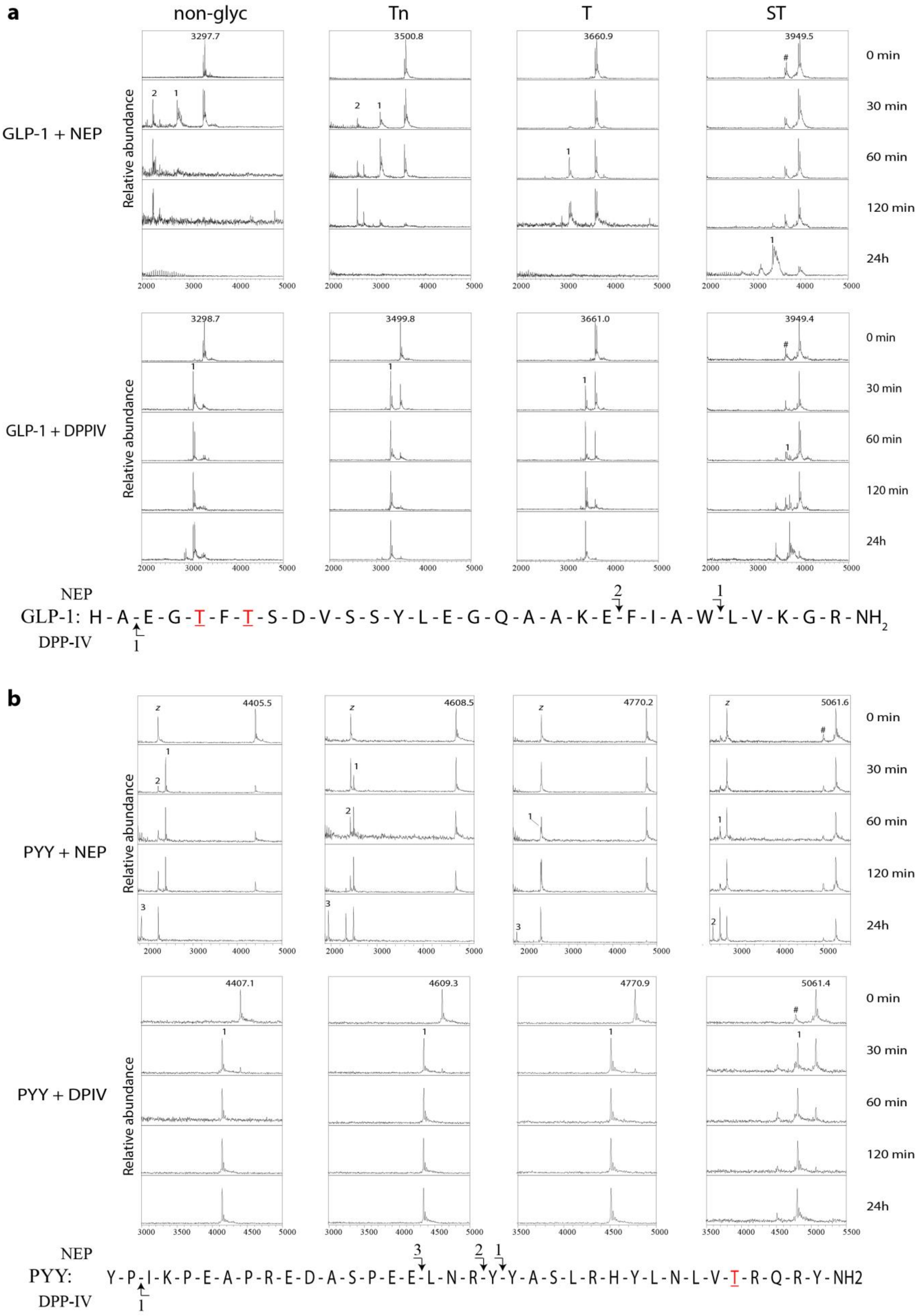

Supplementary Figure 8 continued.

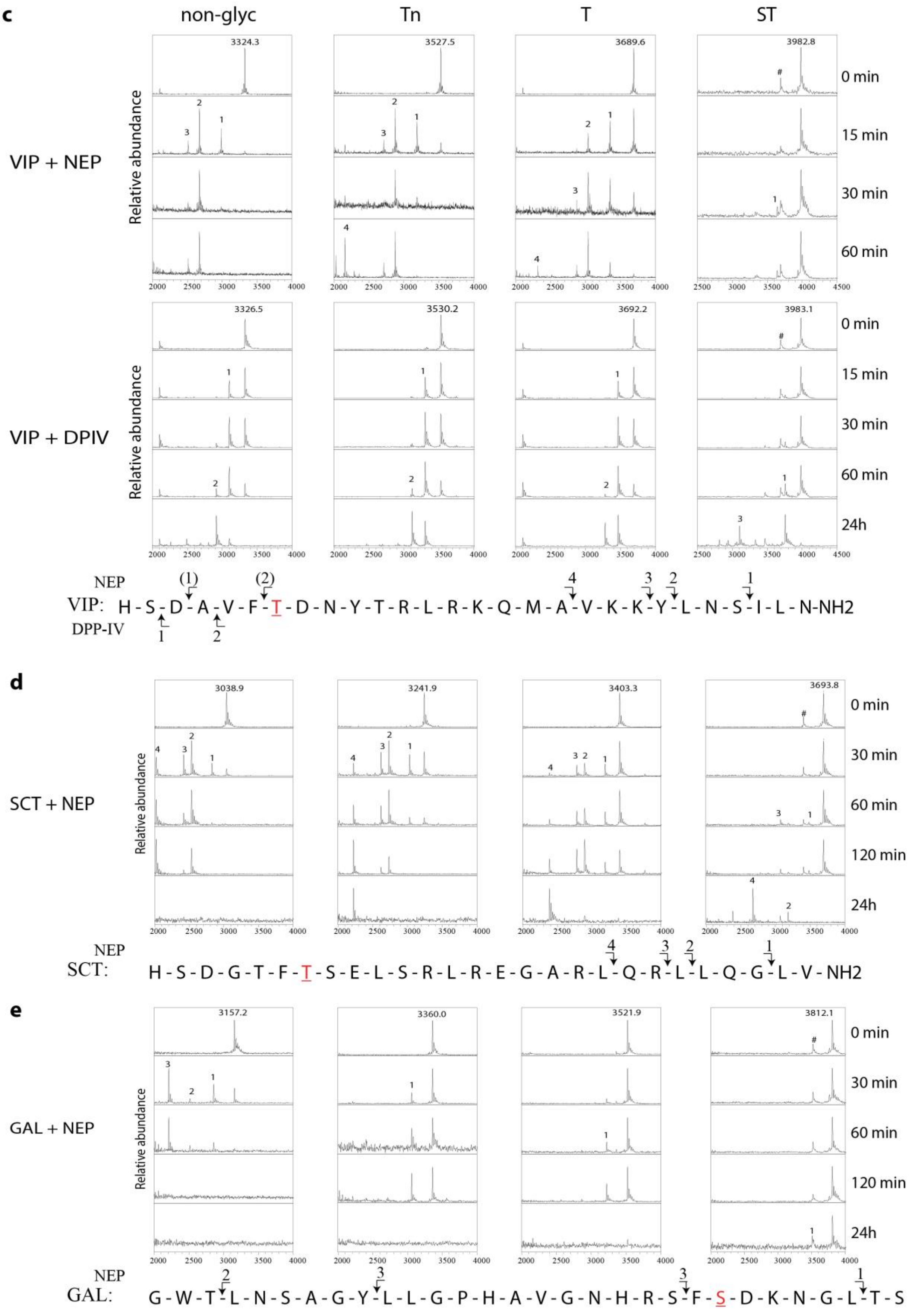

Supplementary Figure 8. Proteolytic stability of glycosylated peptide hormone. *In vitro* cleavage analysis of equimolar non-glycosylated peptide hormone and O-glycosylated hormones treated with either neprilysin (NEP) or dipeptidyl peptidase (DPP-IV) enzyme in a time course of up to 24 hrs. Peptide sequences are shown below each panel with glycosites in red font and observed processing sites of NEP and DPP-IV enzymes are indicated by arrows with numbers corresponding to degradation product peaks in the spectrum. Numbers in parenthesis show possible alternative cleavage products with indistinguishable mass from the indicated processing site. Sample aliqouts were measured at the indicated time points, and product development was monitored by MALDI-TOF mass spectrometry. z denotes doubly charged state of non-degraded peptide. Due to in-source decay of ST-peptides, a small amount of T-glycosylated peptide is visible (#). Panels show **a)** GLP-1$^{Thr5/Thr7}$ (a mixture of monoglycosylated Thr5 and monoglycosylated Thr7); **b)** PYY^{Thr32} **c)** VIP^{Thr7} **d)** SCT^{Thr7}, **e)** GAL^{Ser23}.

Supplementary Table 1. EC50 and extrapolated efficacies of peptide hormones and their glycoforms on cognate receptors.

Agonist	NPY1R Efficacy (%)	NPY1R $Log(EC_{50})$	NPY1R EC_{50} (nM)	NPY2R Efficacy (%)	NPY2R Log(EC50)	NPY2R EC50 (nM)	NPY4R Efficacy (%)	NPY4R Log(EC50)	NPY4R EC50 (nM)	NPY5R Efficacy (%)	NPY5R Log(EC50)	NPY5R EC50 (nM)
PYY	100 ±12,3	-8,58 ±0,03	2,63	100 ±14,6	-8,37 ±0,22	4,27	100 ±2,4	-6,69 ±0,05	204	100 ±11,1	-7,72 ±0,08	19,1
PYY Tn	N/A	N/A	N/A	96,0 ±19,7	-6,46 ±0,18	347	N/A	N/A	N/A	109,2 ±4,1	-6,3 ±0,04	501
PYY T	N/A	N/A	N/A	N/A	N/A	N/A	N/A	N/A	N/A	N/A	N/A	N/A
PYY ST	N/A	N/A	N/A	N/A	N/A	N/A	N/A	N/A	N/A	N/A	N/A	N/A
NPY	100 ±4,6	-9,32 ±0,06	0,48	100 ±12,0	-9,46 ±0,06	0,35	100 ±17,6	-7,97 ±0,08	10,7	100 ±15,6	-7,92 ±0,06	12,0
NPY Tn	N/A	N/A	N/A	92,1 ±10,9	-7,24 ±0,20	57,5	N/A	N/A	N/A	86,2 ±17,0	-6,26 ±0,04	550
NPY T	N/A	N/A	N/A	245,4 ±103	-6,01 ±0,10	977	N/A	N/A	N/A	N/A	N/A	N/A
NPY ST	N/A	N/A	N/A	N/A	N/A	N/A	N/A	N/A	N/A	N/A	N/A	N/A

Glycoform tested	GCGR + GCG Efficacy (%)	GCGR + GCG $Log(EC_{50})$	GCGR + GCG EC_{50} (nM)	GLP1R + GLP-1 Efficacy (%)	GLP1R + GLP-1 Log(EC50)	GLP1R + GLP-1 EC50 (nM)	VPAC1 + VIP Efficacy (%)	VPAC1 + VIP Log(EC50)	VPAC1 + VIP EC50 (nM)	VPAC2 + VIP Efficacy (%)	VPAC2 + VIP Log(EC50)	VPAC2 + VIP EC50 (nM)
Non-glycosylated	100,1 ±0,08	-8,87 ±0,05	1,3	99,5 ±0,5	10,28 ±0,05	0,05	100 ±0,00	-9,72 ±0,07	0,19	100 ±0,01	-9,37 ±0,11	0,42
Tn	106,3 ±12,8	-6,93 ±0,06	116	108 ±6,4	-8,41 ±0,07	3,9	106,3 ±2,6	-6,99 ±0,06	100	112,9 ±36,55	-6,25 ±0,07	551
T	147,2 ±34	-6,21 ±0,09	610	119,8 ±11,8	-6,74 ±0,08	182	103,2 ±3,6	-7,33 ±0,06	46	82,9 ±11,9	-6,35 ±0,10	439
ST	83,9 ±30,8	-5,96 ±0,10	1084	112,5 ±11	-6,65 ±0,05	223	99,0 ±6,7	-7,03 ±0,05	92	73,8 ±0,91	-6,20 ± 0,07	630

Supplementary Table 1. All experiments were performed using COS-7 cells transiently transfected with the indicated receptor. $Log(EC_{50})$, Efficacy (E_{max}) and standard error (±S.E.M) were calculated by nonlinear fitting (three parameter logistic fit with hill coefficient = 1) either without constraint (for efficacy extrapolations) or with E_{max} constrained at 100% receptor activation (for $log(EC_{50})$ calculations). 100% receptor activation is determined as the maximal response obtainable with the non-glycosylated agonist.
