# Supplementary material for: An atlas of O-linked glycosylation on peptide hormones reveals diverse biological roles
Source: Nat Commun. 2020 Aug 20;11:4033. doi: 10.1038/s41467-020-17473-1 (PMC7441158; doi:10.1038/s41467-020-17473-1)
Supplement: Supplementary file 3 — Description of Additional Supplementary Files [file 41467_2020_17473_MOESM3_ESM.docx]

**Description of Supplementary Files**

**File name:** Supplementary data 1.

**Description:** Atlas of glycosites found on peptide hormones in different tissues.

**File name:** Supplementary data 2.

**Description:** Summary of different extraction methods used in this study. Top list summarized the number of peptide hormones per biosource/extraction combination, and bottom list presents which conditions.

**File name:** Supplementary data 3.

**Description:** LC-MS sequenced glycopeptide-spectrum match (PSM) covering the glycosites on each peptide hormone.

**File name:** Supplementary data 4.

**Description:** Examples of extracted spectra for each peptide hormone identified with a glycan.

**File name:** Supplementary data 5.

**Description:** Previously published phosphosites covering peptide hormones.
