# Supplementary material for: An atlas of O-linked glycosylation on peptide hormones reveals diverse biological roles
Source: Nat Commun. 2020 Aug 20;11:4033. doi: 10.1038/s41467-020-17473-1 (PMC7441158; doi:10.1038/s41467-020-17473-1)
Supplement: Supplementary file 9 — Reporting Summary [file 41467_2020_17473_MOESM9_ESM.pdf]

nature research

Corresponding author(s): Katrine T. Schjoldager

Last updated by author(s): Jun 17, 2020

# Reporting Summary

Nature Research wishes to improve the reproducibility of the work that we publish. This form provides structure for consistency and transparency in reporting. For further information on Nature Research policies, see Authors & Referees and the Editorial Policy Checklist .

## Statistics

For all statistical analyses, confirm that the following items are present in the figure legend, table legend, main text, or Methods section.

| n/a | Confirmed | |
|---|---|---|
| ☐ | ☒ | The exact sample size (*n*) for each experimental group/condition, given as a discrete number and unit of measurement |
| ☐ | ☒ | A statement on whether measurements were taken from distinct samples or whether the same sample was measured repeatedly |
| ☐ | ☒ | The statistical test(s) used AND whether they are one- or two-sided<br>*Only common tests should be described solely by name; describe more complex techniques in the Methods section.* |
| ☐ | ☒ | A description of all covariates tested |
| ☐ | ☒ | A description of any assumptions or corrections, such as tests of normality and adjustment for multiple comparisons |
| ☐ | ☒ | A full description of the statistical parameters including central tendency (e.g. means) or other basic estimates (e.g. regression coefficient) AND variation (e.g. standard deviation) or associated estimates of uncertainty (e.g. confidence intervals) |
| ☐ | ☒ | For null hypothesis testing, the test statistic (e.g. *F*, *t*, *r*) with confidence intervals, effect sizes, degrees of freedom and *P* value noted<br>*Give P values as exact values whenever suitable.* |
| ☒ | ☐ | For Bayesian analysis, information on the choice of priors and Markov chain Monte Carlo settings |
| ☒ | ☐ | For hierarchical and complex designs, identification of the appropriate level for tests and full reporting of outcomes |
| ☒ | ☐ | Estimates of effect sizes (e.g. Cohen's *d*, Pearson's *r*), indicating how they were calculated |

*Our web collection on statistics for biologists contains articles on many of the points above.*

## Software and code

Policy information about availability of computer code

| | |
|---|---|
| Data collection | For EASY-nLC 1000 Fusion data acquisition: Thermo Scientific Xcalibur v4.2.47<br>For EASY-nLC 1000 Velos Pro data acquisition: Thermo Scientific Xcalibur v3.0.63<br>For MALDI data acquisition: flexControl v3.4 (Bruker Daltonik GmbH ©)<br>For CD spectroscopy data acquisition: Jasco Spectra Manager v1.53.01<br>For Western blot image acquisition: ImageQuantTM LAS 4000, v1.2<br>For receptor activation assays, data was acquired using Perkin Elmer EnVision Software v1.12<br>For RIA measurements: RiaSmartTM software. |
| Data analysis | Analysis of data from liquid chromatography-tandem mass spectrometry was performed with Proteome Discoverer v1.4<br>For MALDI analysis: FlexAnalysis v3.4 (Bruker Daltonik GmbH ©)<br>For statistical analysis all parameters and errors were calculated using GraphPad Prism version 8.4.2.<br>For half-life calculations R version 3.6.0 (2019-04-26) and R Studio version 1.2.1335 was used.<br>For prediction of O-glycosylation sites we used NetOGlyc 4.0.0.15 software. |

For manuscripts utilizing custom algorithms or software that are central to the research but not yet described in published literature, software must be made available to editors/reviewers. We strongly encourage code deposition in a community repository (e.g. GitHub). See the Nature Research guidelines for submitting code & software for further information.

## Data

Policy information about availability of data

All manuscripts must include a data availability statement. This statement should provide the following information, where applicable:

- Accession codes, unique identifiers, or web links for publicly available datasets
- A list of figures that have associated raw data
- A description of any restrictions on data availability

All mass spectrometry based raw data underlying Figure 1, Table 1, Figure 2, Figure 3, Figure S1, S2 and S3 have been deposited to the ProteomeXchange Consortium via the MassIVE partner repository with the dataset identifier PXD018560 [http://proteomecentral.proteomexchange.org/cgi/GetDataset?ID=PXD018560]. Data is also available at the MassIVE repository with the dataset identifier [MSV000085289]. All other data underlying the findings in this study are available upon request. In addition to the glycoproteomic data, a Source Data file is included containing data underlying all other figures (Figure 4, supplementary figure 4-8).

# Field-specific reporting

Please select the one below that is the best fit for your research. If you are not sure, read the appropriate sections before making your selection.

☒ Life sciences ☐ Behavioural & social sciences ☐ Ecological, evolutionary & environmental sciences

For a reference copy of the document with all sections, see nature.com/documents/nr-reporting-summary-flat.pdf

# Life sciences study design

All studies must disclose on these points even when the disclosure is negative.

**Sample size**

Sample size was chosen based on experience of the investigators with similar experiments conducted multiple times and previously published.

**Data exclusions**

No data was excluded from the analysis, apart from half-life analysis (Fig. 6b), as stated in the main text "For each glycoform, timepoints represented by n>4 measurements were included in the analysis" to ensure proper representation of the mean. All data for Fig. 6b including excluded data points below or above assay detection range are summarised in the source file.

**Replication**

Each tissue or biofluid glycoproteome analysis was performed with 2-3 different extraction methods and in some cases 2 different digestion strategies (except for STC-1 cell line). All attempts were successful.
The receptor activation experiments were repeated 3-5 times in duplicates with consistent results. All attempts were successful as evaluated by the measurement of EC50 values for non-glycosylated agonists in agreement with the literature.
The CD-spectroscopy was performed with each peptide or glycopeptide (n=3 or n=4) repeated on different days also showing consistent results. All attempts were successful.
For the assays exploring peptide stability enzyme kinetics were optimized using non-glycosylated peptide (n=2-3).
Western blots using anti-NPY were repeated (n=3) with similar results obtained.

**Randomization**

For the experiment in Fig. 4d cells receiving glycopeptide or non-glycosylated peptide were selected randomly.
For the experiment described in Fig. 5b, the administration of drug (peptide vs glycopeptide) to the two groups of animals was randomly selected, except that two animals in the same cage did not receive same drug.

**Blinding**

For the experiment described in Fig. 5b the data acquisition was automated and the data processing was performed manually after decoding the samples.

# Reporting for specific materials, systems and methods

We require information from authors about some types of materials, experimental systems and methods used in many studies. Here, indicate whether each material, system or method listed is relevant to your study. If you are not sure if a list item applies to your research, read the appropriate section before selecting a response.

## Materials & experimental systems

| n/a | Involved in the study | |
|---|---|---|
| ☐ | ☒ | Antibodies |
| ☐ | ☒ | Eukaryotic cell lines |
| ☒ | ☐ | Palaeontology |
| ☐ | ☒ | Animals and other organisms |
| ☐ | ☒ | Human research participants |
| ☒ | ☐ | Clinical data |

## Methods

| n/a | Involved in the study | |
|---|---|---|
| ☒ | ☐ | ChIP-seq |
| ☒ | ☐ | Flow cytometry |
| ☒ | ☐ | MRI-based neuroimaging |

## Antibodies

| | |
|---|---|
| Antibodies used | Anti-NPY, Cataglog: D7Y5A, Ref:10/2017, Cell Signaling Technology. Antiserum GLP1, produced in house (assay code: 89390), described and characterized in Ørskov et al., 1994 DOI: 10.2337/diab.43.4.535. |
| Validation | The anti-NPY antibody cross reacts with human, mouse and rat but not with related familiy members PYY,PPY (Ørskov et al., 1994 DOI: 10.2337/diab.43.4.535). Western blot analysis of cells without transfection with preproNPY performed in-house confirms no reactivity. The GLP-1 antibody has been extensively characterised and validated in reference mentioned above. |

## Eukaryotic cell lines

Policy information about cell lines

| | |
|---|---|
| Cell line source(s) | N2A (CCL-131), STC-1 (CRL-3254), COS-7 (CRL-1651) are derived from ATCC, Virginia, USA. HEK293-E cells are obtained through a license agreement with Dr. YVES DUROCHER, Animal Cell Technology and Downstream Processing Group, Biotechnology Research Institute, National Research Council Canada, 6100 Royalmount Avenue, Montreal, Quebec H4P 2R2, Canada |
| Authentication | No specific authentication was used. However all original obtained cell lines were handled separately throughout the entire project. |
| Mycoplasma contamination | All cell lines were tested negative for mycoplasma infection. |
| Commonly misidentified lines (See ICLAC register) | No commonly misidentified cell lines were used in this study. |

## Animals and other organisms

Policy information about studies involving animals; ARRIVE guidelines recommended for reporting animal research

| | |
|---|---|
| Laboratory animals | For the GLP-1 in vivo stability experiment described in Figure 4i, 10 weeks old male Wistar rats were used. Porcine tissues were extracted from female pigs of the LYD strain weighing approximately 30 kg. |
| Wild animals | The study did not involve wild animals |
| Field-collected samples | The study did not involve samples collected in the field. |
| Ethics oversight | Rat in vivo studies and the extraction of porcine tissues were conducted with permission from the Danish Animal Experiments Inspectorate (2013-15-2934-00833) and the in accordance with the guidelines of Danish legislation governing animal experimentation (1987) and the National Institutes of Health (publication number 85-23). |

Note that full information on the approval of the study protocol must also be provided in the manuscript.

## Human research participants

Policy information about studies involving human research participants

| | |
|---|---|
| Population characteristics | As previously described in Nielsen et al., APMIS 2009 (DOI: 10.1111/j.1600-0463.2008.00016.x); Prostate tissue specimens were collected from 25 patients undergoing radical prostatectomy for cancer [median age 65 years (range 54–72)]. |
| Recruitment | All patients had T1-2/Nx-N0/M0 tumors; with Gleason scores ranging from 6 to 9, the median preoperative tPSA was 9.6 mg/l (1.2–52.0). None of the patients were treated with hormonal drugs before surgery. Patients subjects were all Caucasian. |
| Ethics oversight | The Regional Committee on Health Research Ethics approved the use of human tissue (KF 01287197), written informed consent was obtained from all study participants, and the study abides by the Declaration of Helsinki principles.<br><br>Plasma and CSF samples were collected with written informed concent and subsequently pooled and anonymized. The National Committee on Health Research Ethics has evaluated that the use of the plasma and CSF samples for the glycoproteomics study did not need approval from the committee because of the anonymization of patient samples. |

Note that full information on the approval of the study protocol must also be provided in the manuscript.
